# Higher Reduced State of Fe/S-Signals, with the Suppressed Oxidation of P700, Causes PSI Inactivation in *Arabidopsis thaliana*

**DOI:** 10.3390/antiox12010021

**Published:** 2022-12-22

**Authors:** Riu Furutani, Shinya Wada, Kentaro Ifuku, Shu Maekawa, Chikahiro Miyake

**Affiliations:** 1 Graduate School for Agricultural Science, Kobe University, 1-1 Rokkodai, Nada-ku, Kobe 657-8501, Japan; 2 Core Research for Evolutional Science and Technology (CREST), Japan Science and Technology Agency (JST), 7 Gobancho, Tokyo 102-0076, Japan; 3 Graduate School for Agriculture, Kyoto University, Kitashirakawa Oiwake-cho, Sakyo-ku, Kyoto 606-8502, Japan

**Keywords:** Fe/S clusters, ferredoxin, photosynthetic electron transport, photosystem I, photoinhibition, P700

## Abstract

Environmental stress increases the risk of electron accumulation in photosystem I (PSI) of chloroplasts, which can cause oxygen (O_2_) reduction to superoxide radicals and decreased photosynthetic ability. We used three *Arabidopsis thaliana* lines: wild-type (WT) and the mutants *pgr5^hope1^* and *paa1*-*7*/*pox1*. These lines have different reduced states of iron/sulfur (Fe/S) signals, including F_x_, F_A_/F_B,_ and ferredoxin, the electron carriers at the acceptor side of PSI. In the dark, short-pulse light was repetitively illuminated to the intact leaves of the plants to provide electrons to the acceptor side of PSI. WT and *pgr5^hope1^* plants showed full reductions of Fe/S during short-pulse light and PSI inactivation. In contrast, *paa1*-*7*/*pox1* showed less reduction of Fe/S and its PSI was not inactivated. Under continuous actinic-light illumination, *pgr5^hope1^* showed no P700 oxidation with higher Fe/S reduction due to the loss of photosynthesis control and PSI inactivation. These results indicate that the accumulation of electrons at the acceptor side of PSI may trigger the production of superoxide radicals. P700 oxidation, responsible for the robustness of photosynthetic organisms, participates in reactive oxygen species suppression by oxidizing the acceptor side of PSI.

## 1. Introduction

Oxygen (O_2_) in the atmosphere exists as triplet molecules (^3^O_2_) that have two unpaired electrons. Therefore, O_2_ easily reacts with radicals and molecules with low redox potential from which it receives electrons, resulting in the production of superoxide radicals (O_2_^−^) [1]. Superoxide radicals disproportionate to hydrogen peroxide (H_2_O_2_) and water (H_2_O), and H_2_O_2_ can further produce hydroxyl radicals (·OH) via a Fenton reaction with reduced iron, i.e., Fe(II). Reactive oxygen species (ROS), such as O_2_^−^, H_2_O_2_, and OH, are highly reactive and can easily oxidize DNA, proteins, and lipids in cells [2,3,4].

In the photosynthetic electron transport reaction, and depending on the difference in the redox potential of the sequential electron carriers, the electrons produced in photosystem (PS) II are transported from the acceptor side of PSII to the donor side of PSI through plastoquinone, the cytochrome (Cyt) *b*6/*f*-complex, and plastocyanin (PC), and from the PSI acceptor side to NADP^+^ through phylloquinone A_1_, iron/sulfur (Fe/S) clusters, namely F_x_ and F_A_/F_B_, and ferredoxin (Fd). In PSI, the redox potential of these electron carriers is sufficiently low to reduce O_2_ to O_2_^−^ [4,5]. The production of H_2_O_2_ in PSI was first observed by Mehler [6,7] and Mehler and Brown [8]. Thereafter, Asada and Kiso [9] and Asada et al. [10] identified the primary product of the O_2_ reduction reaction in PSI to be O_2_^−^. Furthermore, Takahashi and Asada [11] found that O_2_ can be reduced to O_2_^−^ by Fe/S in PSI, and both Kozuleva et al. [12,13] and Kruk [14] reported that phylloquinone can reduce O_2_ to O_2_^−^. Khorobrykh et al. [4] suggested the production of ·OH by the reaction of O_2_^−^ with H_2_O_2_ catalyzed by Fe/S in PSI.

The in vivo production of ROS in PSI can be inferred from the fact that PSI inactivation occurs with the dependency of O_2_ in intact leaves. Repetitive short-pulse (rSP) light illumination of intact leaves under dark conditions induces electron accumulation in the photosynthetic electron transport system [15,16]. During rSP light illumination, PSI, but not PSII, was inactivated, causing a decrease in the carbon dioxide (CO_2_) assimilation rate. In addition, the inactivation of PSI depends on atmospheric O_2_ concentration [15]. Takahashi and Asada [11,17,18] reported that short-pulse light illumination produced O_2_^−^ in the PSI of thylakoid membranes in vitro. Furthermore, the ROS produced in PSI may directly and immediately inactivate PSI, inducing oxidative damage to PSI proteins.

In the present study, we elucidated the relationship between electron accumulation at the acceptor side of PSI and inactivation of PSI in intact leaves of *Arabidopsis thaliana*. We monitored the reduction-oxidation (redox) states of P700 and Fe/S, including F_x_ and F_A_/F_B_, and Fd in PSI, using the intact leaves of wild-type (WT) and mutant *pgr5^hope1^* and *paa1*-*7*/*pox1 A. thaliana*, which exhibit suppressed and enhanced oxidation levels of P700 [19,20]. These mutants were expected to have different electron accumulation levels on the acceptor side of PSI. We found that a higher reduced state (more electron accumulation) of both Fe/S and Fd accelerated the inactivation rate of PSI. The molecular mechanism of ROS production in PSI and the physiological function of P700 oxidation to suppress PSI inactivation in vivo are discussed.

## 2. Materials and Methods

### 2.1. Plant Materials and Growth Conditions

WT and mutant (*pgr5^hope1^* and *paa1*-*7*/*pox1*) *A. thaliana* (gl-1) were grown in soil pots containing a 2:1.5 ratio of seeding-culture soil (TAKII Co., Ltd., Kyoto, Japan) to vermiculite. The *pgr5^hope1^* and *paa1*-*7*/*pox1* ethyl methane sulfonate-induced mutants originated from Wada et al. [20] and Furutani et al. [21], respectively. The pots were placed in a controlled chamber (14 h light at 23 °C/10 h darkness at 20 °C; photon flux density: 100–150 µmol photons m^−2^ s^−1^; relative humidity: 55–60%). Seeds were planted in the soil after 3 days of vernalization at 4 °C. The plants were watered every 2–3 days, and 1000-fold diluted Hyponex solution (Hyponex, Osaka, Japan) was applied weekly after seeding. Measurements were conducted using rosette leaves of 4–5 week old plants.

### 2.2. Simultaneous Measurements of Chlorophyll (Chl) Fluorescence, P700, and Fe/S-Signals with Gas-Exchange

Chl fluorescence, P700, Fe/S, including F_x_, F_A_/F_B_, and Fd, and CO_2_ exchange were simultaneously measured using Dual/KLAS-NIR [22,23] (Heinz Walz GmbH, Effeltrich, Germany) and infra-red gas analyzer (IRGA) LI-7000 (Li-COR, Lincoln, NE, USA) measuring systems equipped with a 3010-DUAL gas exchange chamber at 40 Pa CO_2_/21 kPa O_2_ (Heinz Walz GmbH). The gases were saturated with water vapor at 16 ± 0.1 °C. The leaf temperature was controlled at 25 ± 0.5 °C (relative humidity: 55–60%). The actinic photon flux density at the upper position on the leaf in the chamber was adjusted to the indicated intensity. The net CO_2_ assimilation rate (A) and the dark respiration rate (Rd) were measured. The Chl fluorescence parameters were calculated [24] as follows: maximum quantum efficiency of PSII photochemistry, *F*_v_/*F*_m_ = (*F*_m_ − *F*_o_)/*F*_m_; *F*_o_, minimum fluorescence yield; *F*_m_, maximum fluorescence yield. The signals for the oxidized P700 (P700^+^) and reduced Fe/S (Fe/S^−^) were calculated based on the deconvolution of four pulse-modulated dual-wavelength difference signals in the near-infrared region (780–820, 820–870, 840–965, and 870–965 nm) [22]. P700 was completely reduced and Fe/S was fully oxidized in the dark. To determine the total photo-oxidizable P700 (Pm), a saturation flash was applied after 10 s of illumination with far-red light (740 nm). Total photo-reducible Fe/S was determined by illumination with red actinic light (450 µmol photons m^−2^ s^−1^) after plant leaves were adapted to the dark for 5 min [22]. The redox state of P700 under actinic light illumination was evaluated as the ratio of P700^+^ to total P700. The incident photo-oxidizable P700 obtained by short-pulse light during rSP light illumination treatment was termed Pm’ [15].

### 2.3. Constant High-Intensity Light Treatment

High-intensity light treatments of both WT and *pgr5^hope1^* plants were conducted under ambient air conditions (40 Pa CO_2_ and 21 kPa O_2_). After 15 min of photosynthesis induction (AL, 550 µmol photons m^−2^ s^−1^), the leaves were exposed to high-intensity light (1100 µmol photons m^−2^ s^−1^) for 120 min. To minimize the effect of rSP light illumination on PSI photoinactivation, the photosynthetic parameter Pm’ was recorded every 15 min [15]. The leaves were left in the dark for 30 min after treatment, and then the *F*_v_/*F*_m_ of PSII, Pm, and magnitude of Fe/S were measured.

### 2.4. Statistical Analysis

Statistical analysis, Welch’s *t*-test, which are included in Microsoft Excel for Mac (ver. 16.16.27), were performed to detect any significant differences (* *p* < 0.05, ** *p* < 0.01, *** *p* < 0.001).

## 3. Results

We have previously reported that rSP light illumination induces inactivation of PSI in intact leaves of plants under atmospheric O_2_ conditions [15,25,26]. Comparing the ratio of PSI inactivation among land plants (liverworts, ferns, gymnosperms, and angiosperms), angiosperms suffered the most severe PSI inactivation [26]. This is because liverworts, ferns, and gymnosperms have flavodiiron proteins (FLV) in their chloroplasts, which produce H_2_O by reducing O_2_ using the reducing power (electrons) from the photosynthetic electron transport system, but angiosperms do not. FLV function as electron acceptors from PSI even during short-pulse light (~1000 ms). Therefore, P700 is reduced during short-pulse illumination in angiosperms, while in other plants FLV oxidize P700. This indicates that in angiosperms, including Arabidopsis, the oxidation reaction of the excited P700 to the oxidized form, P700^+^, is limited during short-pulse light, and electrons should accumulate at the acceptor side of PSI. Simultaneously, the accumulated electrons can flow to O_2_ producing O_2_^−^. To confirm electron accumulation at the acceptor side of PSI during short-pulse light, we monitored Fe/S, representing both the Fe/S clusters and Fd, in PSI using DUAL/KLAS-NIR [23,27,28].

**Figure 1:**
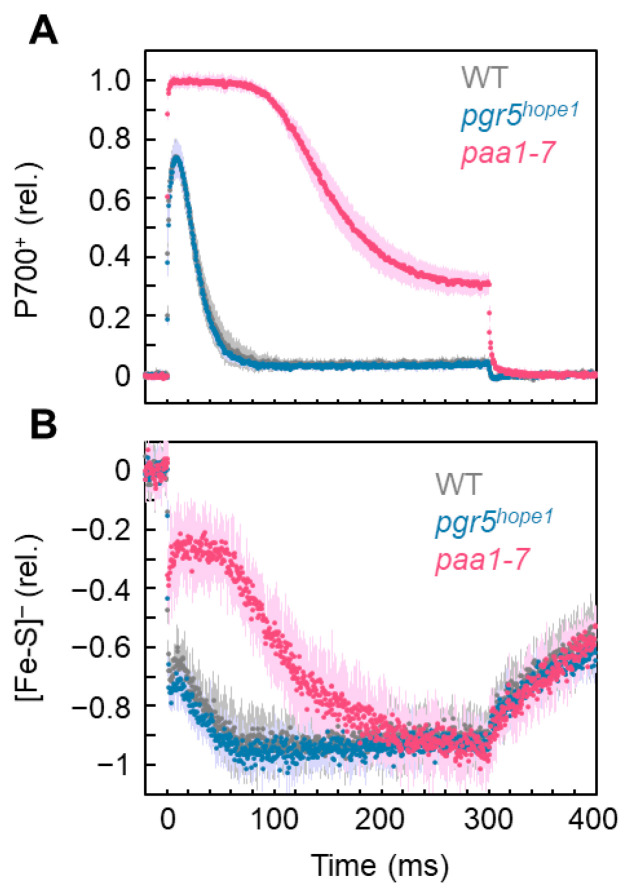
Kinetics of (**A**) oxidized P700 (P700^+^) and (**B**) reduced Fe/S signals (Fe/S^−^) in response to short-pulse light for wild-type (WT), *pgr5^hope1^*, and *paa1-7* Arabidopsis intact leaves. Short-pulse light (15,000 µmol photons m^−2^ s^−1^, 300 ms) was started at 0 ms. The redox reactions of both P700 and Fe/S were monitored simultaneously. Relative values of both P700^+^ and [Fe/S]^−^ were normalized to the maximum oxidation and reduction levels, respectively, as described in the “Materials and Methods”. The negative values of Fe/S^−^ show the reduction of Fe/S. The data points for WT (gray), *pgr5^hope1^* (magenta), and *paa1*-*7*/*pox1* (red) are the means of six biological replicates (darker color) and the shadowed area is the standard deviation (lighter color).

Upon illumination with short-pulse light (300 ms, 15,000 µmol photons m^−2^ s^−1^) of WT *A. thaliana* dark-adapted leaves, P700 was rapidly oxidized to P700^+^, achieving its maximum value at approximately 10 ms, and then decreased to its minimum at approximately 100 ms (Figure 1A). Upon illumination with short-pulse light, Fe/S was reduced to 70%, then transiently oxidized to 65%, and thereafter largely reduced to 100%. As shown in Figure 1B, the time range of the second reduction of Fe/S corresponds to the reduction of P700^+^ after reaching its maximum oxidation. That is, the reduction of P700^+^ after approximately 10 ms of short-pulse light resulted in the accumulation of electrons in the Fe/S of PSI. The maximum accumulation of electrons in Fe/S suppressed P700^+^ to its minimum, indicating the limitation of the oxidation reaction of the excited P700 during short-pulse light. The Arabidopsis mutant *pgr5^hope1^* has an identical point mutation in *PGR5* gene (At2g05620) as the *pgr5-1* mutant and is therefore deficient in PGR5 protein [19,20]. *pgr5^hope1^* has shown no P700 oxidation under continuous illumination with actinic light [19,20]. Compared to the WT, *pgr5^hope1^* showed a lower proton motive force and proton gradient (ΔpH) across the thylakoid membranes but this did not suppress the plastoquinol oxidation activity of the Cyt *b*6/*f* complex. However, photosynthesis control does not function in *pgr5^hope1^* [20]. In the present study, *pgr5^hope1^* did not show any limitation in the reduction reaction of P700^+^ in the photo-oxidation reduction cycle of P700 in PSI. The illumination of short-pulse light to the dark-adapted leaves of *pgr5^hope1^* and the behaviors of P700^+^ and Fe/S were all similar to those of the WT (Figure 1). In contrast to continuous illumination by actinic light, short-pulse light did not form a ΔpH across the thylakoid membranes [29], which did not induce photosynthesis control. Therefore, we could not find any difference in the behaviors of P700^+^ and Fe/S between WT and *pgr5^hope1^* plants under short-pulse light illumination.

The Arabidopsis mutant *paa1-7*/*pox1* was isolated when screening for the P700 oxidation response under single short-pulse illumination; it was identified as a mutant of P-type ATPase, *PAA1* (At4g33525), which functions in copper (Cu) ion transport across the chloroplast envelope [21,30]. The *paa1-7*/*pox1*, similar to other *paa1* mutants, showed a lower amount of PC due to the lack of Cu ion transport into chloroplasts [21,30]. We confirmed a much lower amount of PC compared to WT [21]. Furthermore, the reduction reaction of P700^+^ was expected to be limited in the photo-oxidation reduction cycle of P700 in PSI because electron transfer would be suppressed from the Cyt *b*6/*f* complex to P700 in PSI [21]. The illumination of the short-pulse light to the dark-adapted leaves of *paa1-7*/*pox1 A. thaliana* and the behaviors of P700^+^ and Fe/S were compared to those of the WT (Figure 1). During illumination with short-pulse light, P700 was oxidized for approximately 80 ms after reaching its maximum value, which was different from that of the WT (Figure 1A). The reduction rate of P700^+^ after reaching the maximum was slower than that of the WT, and the final oxidation state of P700^+^ at 300 ms was higher than that of the WT. These facts reflect the limitation of P700^+^ reduction during short-pulse illumination owing to the lower amount of PC. Fe/S took approximately 200 ms for full reduction after the start of the short-pulse light (Figure 1B). During the first 100 ms of the short-pulse light, Fe/S was more oxidized in the *paa1-7*/*pox1* mutant than in the WT. Thereafter, Fe/S was slowly reduced, indicating the accumulation of electrons, leading to the reduction of P700^+^ after approximately 80 ms of short-pulse light. The kinetics of Fe/S showed that the electrons flowed from the Cyt *b*6/*f* complex to PSI at a slower rate in *paa1-7*/*pox1* than in the WT.

**Figure 2:**
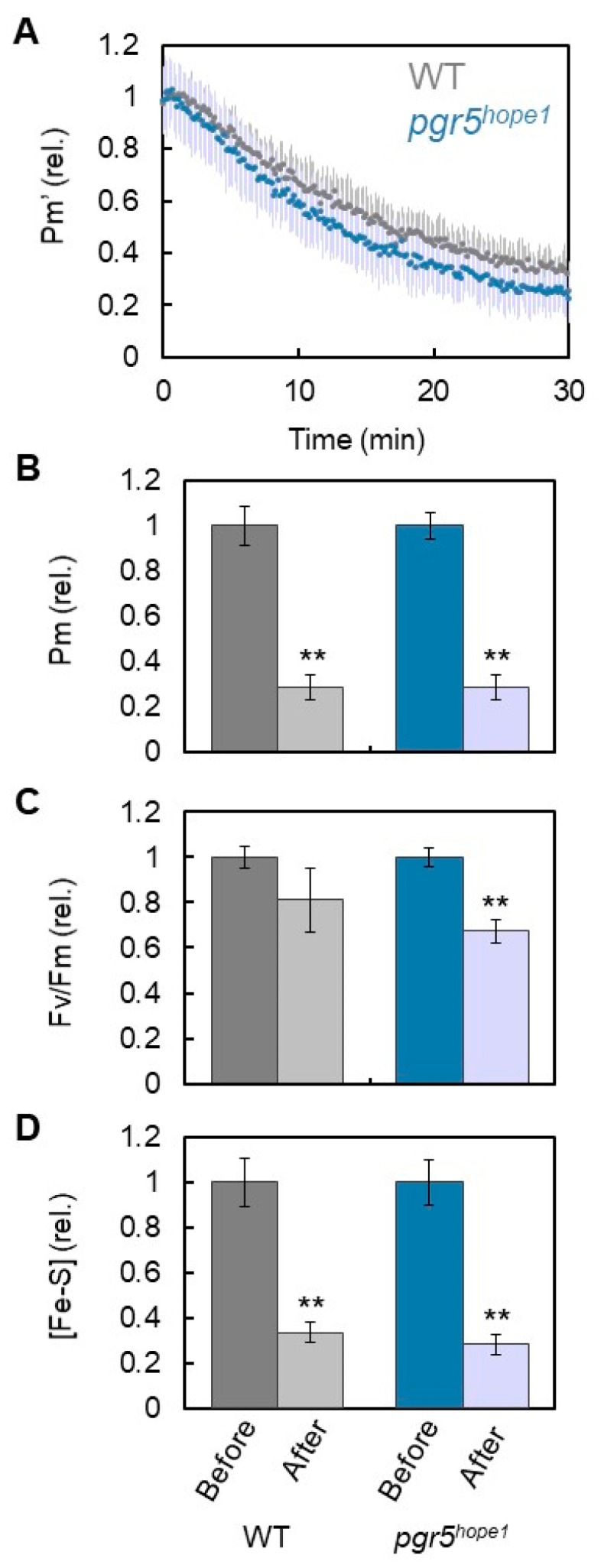
Effects of rSP light illumination on the incident photo-oxidizable P700 (Pm’), photo-oxidizable P700 (Pm), maximum quantum efficiency of PSII (Fv/Fm), and amount of Fe/S in wild-type (WT) and *pgr5^hope1^* Arabidopsis. The leaves were illuminated every 10 s with short-pulse light (300 ms) of 15,000 μmol photons m^−2^ s^−1^ under the atmospheric conditions (40 Pa CO_2_/ 21 kPa O_2_) for 30 min. The rSP light illumination started at 0 min. (**A**) Pm’. Black, WT; magenta, *pgr5^hope1^*. The mean values were normalized to the primary values before the rSP light treatment; error bars represent the standard deviation; data were acquired from six biological replicates. (**B**) Pm, (**C**) Fv/Fm, and (**D**) Fe/S were compared before and after the rSP light illumination treatment in WT and *pgr5^hope1^*. These parameters were evaluated after the illuminated leaves were left for 1 h in the dark. Each value was normalized to the value before the rSP light illumination treatment. ** *p* < 0.01 (Welch’s *t*-test).

Using three plants of WT, *pgr5^hope1^*, and *paa1-7*/*pox1*, we analyzed the effects of electron accumulation at the acceptor side of PSI on PSI inactivation. The accumulation of electrons is represented by the reduction of Fe/S, as shown in Figure 1. During short-pulse light, both WT and *pgr5^hope1^* plants showed the same degree of electron accumulation. In the comparison of WT with *pgr5^hope1^*, the rSP light treatments (300 ms, 15,000 µmol photons m^−2^ s^−1^, every 10 s for 30 min) gradually decreased the Pm’ values in both plant lines (Figure 2A). Pm’ corresponds to the incident photo-oxidizable P700 estimated by short-pulse illumination (see “Materials and Methods”), and a decrease in Pm’ leads to a decrease in Pm. That is, a decrease in Pm’ reflects PSI inactivation [15]. The extent of the Pm’ decrease in WT was similar to that in *pgr5^hope1^*. The residual Pm in both plant lines after 30 min of rSP light treatment was approximately 30% (Figure 2B). In contrast, the residual F_v_/F_m_ of PSII was maintained above 80% in WT and approximately 70% in *pgr5^hope1^*. PSI was largely damaged by short-pulse light compared to PSII and at similar extent in both WT and *pgr5^hope1^*. Furthermore, we found a decrease in Fe/S after the rSP light treatment. The extent of the Fe/S decrease in WT (approximately 30%) was similar to that of *pgr5^hope1^* (Figure 2D). In other words, Pm decreased as Fe/S decreased. This could be due to the lack of difference in electron accumulation in the Fe/S of both WT and *pgr5^hope1^* plant lines during short-pulse light (Figure 1B).

**Figure 3:**
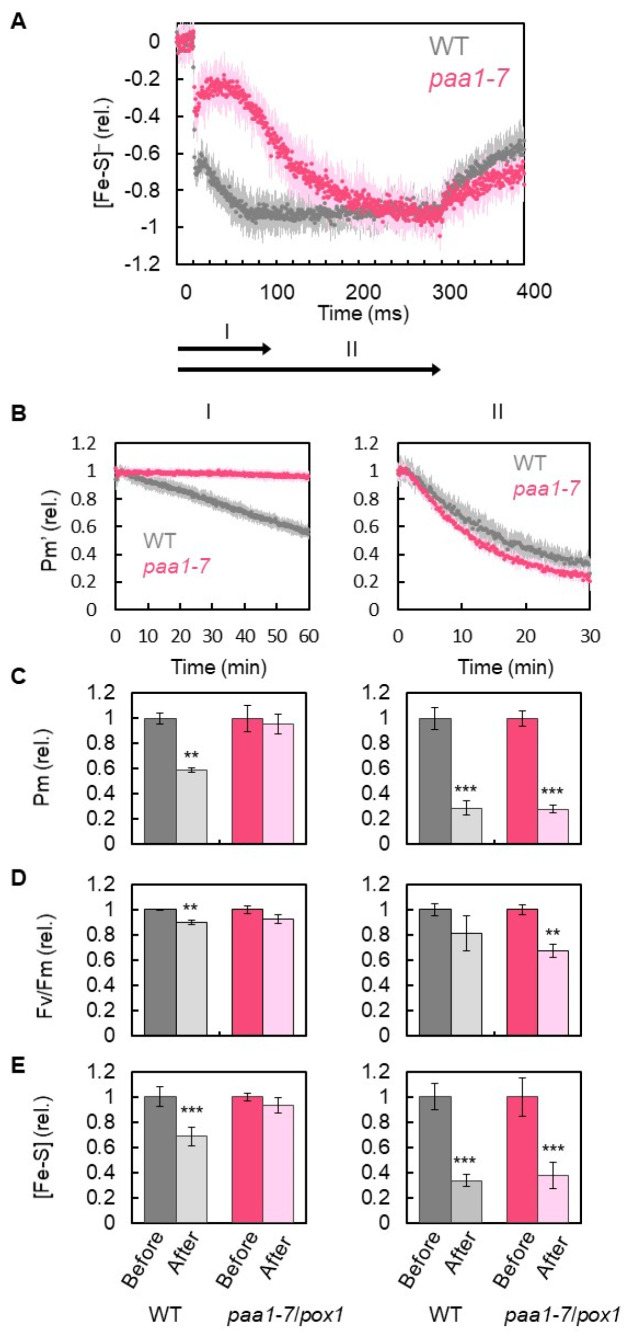
Effects of rSP light illumination and illumination time on the incident photo-oxidizable P700 (Pm’), photo-oxidizable P700 (Pm), maximum quantum efficiency of PSII (Fv/Fm), and amount of Fe/S in wild-type (WT) and *paa1-7/pox1* Arabidopsis. The leaves were illuminated every 10 s with short-pulse light of 15,000 μmol photons m^−2^ s^−1^ under atmospheric conditions (40 Pa CO_2_/ 21 kPa O_2_) for 30 min. The rSP light illumination started at 0 min. (**A**) The illumination time was set to the two durations, as indicated by the arrows (I, 100 ms; II, 300 ms), based on the reduction kinetics of Fe/S of both WT and *paa1-7/pox1* (redrawn from Figure 1, Black, WT; red, *paa1-7/pox1*). In experiments I and II, the parameters Pm’, Pm, Fv/Fm, and [Fe/S]^−^ were analyzed. (**B**) Pm’. Black, WT; magenta, *pgr5^hope1^*. The values were normalized to the primary values before the rSP light illumination treatment and are shown with standard deviations. The data were obtained from six biological replicates. (**C**) Pm, (**D**) Fv/Fm, and (**E**) Fe/S before and after the rSP light illumination treatments in both WT and *pgr5^hope1^* were compared. These parameters were evaluated after the illuminated leaves were left for 1 h in the dark. Each value was normalized against the value before the rSP light illumination treatment. ** *p* < 0.01; *** *p* < 0.001 (Welch’s *t*-test).

The *paa1*-*7*/*pox1* mutant showed two time-based steps of Fe/S reduction: in the first step, the mutant showed less accumulation of electrons during the first 100 ms after the onset of the short-pulse light illumination, as compared to the WT; in the second step, there was full accumulation of electrons after 200 ms. Then, we set the illumination time of the short-pulse light to 100 ms (I) and 300 ms (II) in the rSP light illumination treatments I and II, respectively, for the comparison of PSI inactivation between WT and *paa1*-*7*/*pox1* (Figure 3A). The rSP light illumination treatment I (duration I, 15,000 µmol photons m^−2^ s^−1^, every 10 s for 60 min) applied to WT gradually decreased Pm’; however, the rate of decrease was slower than that of the rSP light illumination treatment II (duration II, 15,000 µmol photons m^−2^ s^−1^, every 10 s for 30 min) (Figure 3B). The residual Pm in rSP light illumination treatment I was approximately 60% at 60 min, whereas it was approximately 30% at 30 min in rSP light illumination treatment II. In contrast to the WT, the rSP light illumination treatment I of *paa1*-*7*/*pox1* did not inactivate PSI and PSII, as reflected by the F_v_/F_m_ (Figure 3C). However, rSP light illumination treatment II inactivated PSI and PSII to the same extent as in the WT (Figure 3C). Furthermore, the residual Fe/S in the rSP light illumination treatment I of WT was approximately 70% at 60 min, whereas it was approximately 30% at 30 min in the rSP light illumination treatment II of WT (Figure 3E). In contrast to the WT, the rSP light illumination treatment I of *paa1*-*7*/*pox1* did not decrease Fe/S (Figure 3D). However, the rSP light illumination treatment II of *paa1*-*7*/*pox1* decreased Fe/S to a similar extent as in the WT (residual Fe/S, about 30%) (Figure 3D).

**Figure 4:**
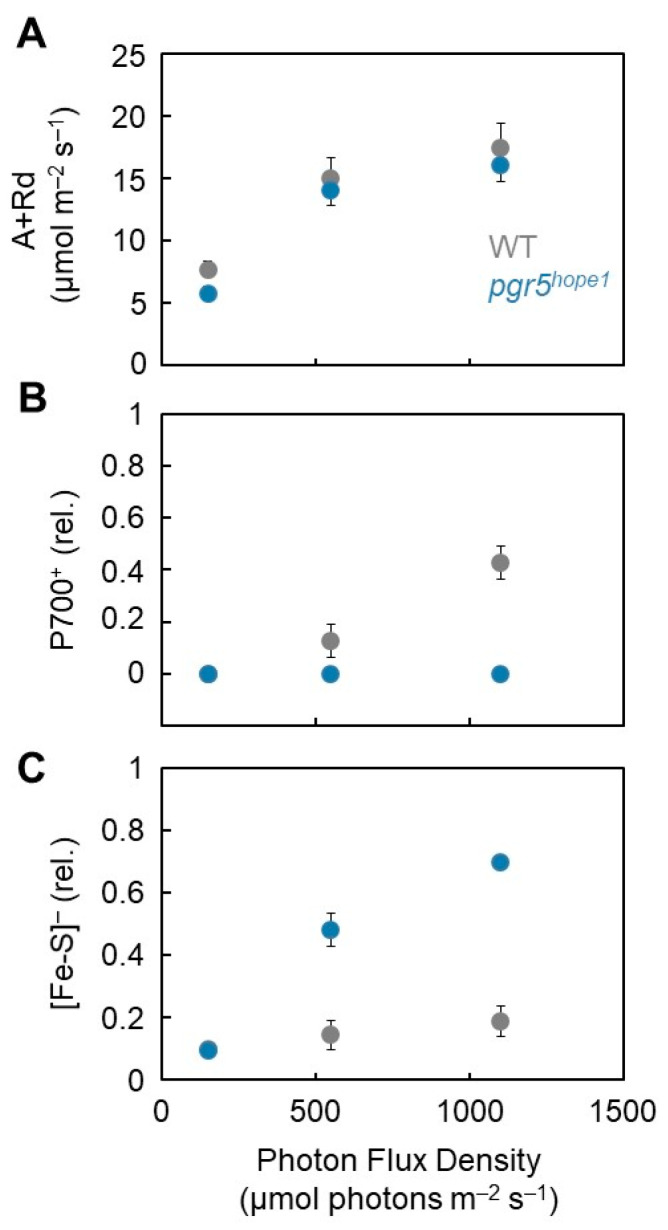
Effect of photon flux density on the gross CO_2_ assimilation rate (A + Rd), oxidized P700 (P700^+^), and reduction ratio of Fe/S signals [Fe/S]^−^ in wild-type (WT) and *pgr5^hope1^* Arabidopsis. (**A**) The net CO_2_ assimilation rates were measured simultaneously with P700^+^ and Fe/S^−^ under atmospheric conditions (40 Pa CO_2_, 21 kPa O_2_). The dark respiration rates (Rd) were measured before starting actinic light illumination. After the net CO_2_ assimilation reached the steady state at the photon flux density of 150 μmol photons m^−2^ s^−1^, the intensity was increased to 550 and 1100 sequentially, after reaching each steady-state CO_2_ assimilation. The gross CO_2_ assimilation rates are expressed as A + Rd. (**B**) The oxidized P700 (P700^+^) is plotted against the photon flux density. (**C**) The reduction ratio of [Fe/S]^−^ is plotted against the photon flux density. The mean of three biological replicates and standard deviation are shown. Gray symbols, WT; Blue symbols, *pgr5^hope1^*.

These results indicate that electron accumulation in Fe/S is the reason for PSI inactivation. The kinetics of both P700^+^ and Fe/S redox reactions during short-pulse light revealed that the preceding reduction of Fe/S and electron accumulation in the acceptor side of PSI induced the reduced state of P700^+^ (Figure 1). Under continuous illumination with actinic light, *pgr5^hope1^* did not show any oxidation of P700^+^ owing to the lack of photosynthesis control, as described above [19,20]. Next, we analyzed the relationship between the redox state of P700 and Fe/S in both WT and *pgr5^hope1^* plants under continuous illumination with actinic light (Figure 4). We compared the dependency of the gross CO_2_ assimilation rate (A + Rd, see “Materials and Methods”) on the photon flux density and found no difference between WT and *pgr5^hope1^* (Figure 4A). However, we found a different dependency of P700 oxidation on the photon flux density (Figure 4B). With an increase in photon flux density, P700 was oxidized to approximately 40% at 1100 μmol photons m^−2^ s^−1^ in WT, but not in *pgr5^hope1^*. Furthermore, we found an enhanced reduction of Fe/S in *pgr5^hope1^* with an increase in the photon flux density compared to the WT (Figure 4C). The reverse relationship between the oxidation of P700 and the reduced state of Fe/S indicated that photosynthesis limits electron donation to P700 in PSI and induces the oxidation of Fe/S.

**Figure 5:**
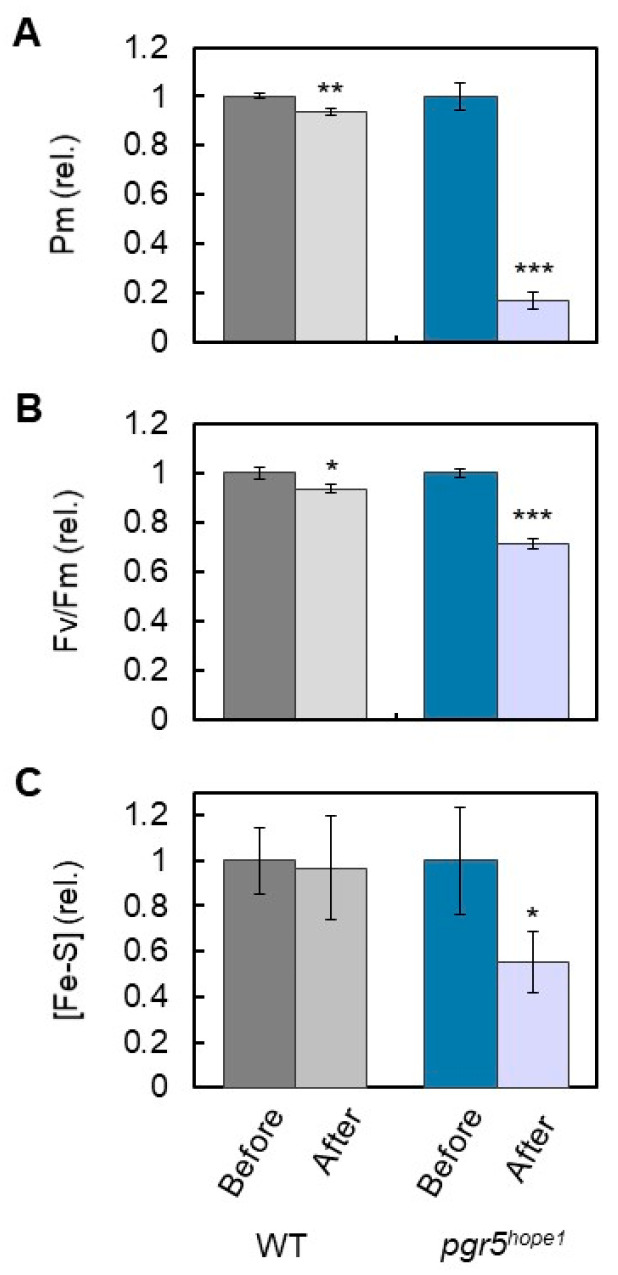
Effects of the continuous illumination of actinic light on the photo-oxidizable P700 (Pm), maximum quantum efficiency of PSII (Fv/Fm), and amount of Fe/S ([Fe/S]) in wild-type (WT) and *pgr5^hope1^* Arabidopsis. After reaching the steady state of the net CO_2_ assimilation rate induced by the continuous illumination of actinic light (500 μmol photons m^−2^ s^−1^, 30 min) in both WT and *pgr5^hope1^*, the photon flux density was increased to 1100 μmol photons m^−2^ s^−1^, and the illumination was continued for 2 h. The parameters, (**A**) Pm, (**B**) Fv/Fm, and (**C**) [Fe/S] of both WT and *pgr5^hope1^* treated with continuous illumination were evaluated after the illuminated leaves were left for 1 h in the dark, and values obtained before and after the continuous light illumination treatment were compared. The mean of three biological replicates and standard deviation are shown. * *p* < 0.05; ** *p* < 0.01; *** *p* < 0.001 (Welch’s *t*-test).

The reduced state of Fe/S, that is, the electron accumulation in Fe/S, might be the cause of PSI inactivation (Figure 2 and Figure 3). After reaching the steady state of the net CO_2_ assimilation rate induced by the continuous illumination of actinic light (500 µmol photons m^−2^ s^−1^, 30 min) in both WT and *pgr5^hope1^*, we increased the photon flux density to 1100 μmol photons m^−2^ s^−1^ and continued to illuminate intact leaves for 2 h. In contrast to the WT, *pgr5^hope1^* showed PSI inactivation (Figure 5A). After the light treatment, Pm decreased to below 20% and Fv/Fm decreased to approximately 70% (Figure 5B). Furthermore, in contrast to the WT, Fe/S decreased to approximately 50% in *pgr5^hope1^*. A positive correlation between electron accumulation in Fe/S and PSI inactivation was therefore confirmed. The electron accumulation in Fe/S could trigger the production of ROS at the acceptor side of PSI and degrade Fe/S and PSI simultaneously.

## 4. Discussion

In the present study, we elucidated the positive relationship between electron accumulation at the acceptor side of PSI and PSI inactivation in *A. thaliana* intact leaves. We used three genotypes of Arabidopsis, which have different reduced states of Fe/S signals, including F_x_, F_A_/F_B_, and Fd in PSI: WT, and the mutants, *pgr5^hope1^* and *paa1*-*7*/*pox1*, which exhibit suppressed and enhanced oxidation levels of P700, respectively [21,30,31,32]. In the dark, where ΔpH across the thylakoid membranes was not induced and photosynthesis control was not activated, rSP light illumination was applied to the intact leaves of plants to provide electrons to the acceptor side of PSI. We confirmed the reduction of Fe/S with the reduction of P700 during short-pulse light in both WT and *pgr5^hope1^* plants and to the same extent. In addition, both plant lines showed PSI inactivation. In contrast, *paa1*-*7*/*pox1* showed two-phase kinetics of Fe/S characterized by slow and fast reductions, which were discriminated by the short-pulse light illumination time. A shorter illumination time (treatment I) reduced Fe/S by less than 25% with the maximum oxidation of P700; a longer illumination time (treatment II) led to the full reduction of Fe/S with a greater reduction in P700. Compared to the WT, the rSP light illumination treatment I did not inactivate the PSI of *paa1*-*7*/*pox1*. The rSP light illumination treatment I inactivated PSI in both WT and *paa1*-*7*/*pox1*. Furthermore, we compared the effects of the reduction of Fe/S on PSI inactivation under continuous illumination with actinic light in both WT and *pgr5^hope1^*. In a previous study, and in contrast to the WT, *pgr5^hope1^* did not induce P700 oxidation [19,20]. This was also observed in the present study. We also found a higher reduction of Fe/S in *pgr5^hope1^* than in the WT. In contrast to *pgr5^hope1^*, photosynthesis control suppressed plastoquinol oxidation activity in the WT, limiting the reduction of oxidized P700 in PSI. We confirmed PSI inactivation in *pgr5^hope1^* plants under continuous illumination with actinic light. These results corresponded to those reported by Wada et al. [20]. The above results evidenced that the higher reduced state of Fe/S in PSI and accumulation of electrons at its acceptor side can trigger the production of ROS, which oxidatively damages PSI.

The higher reduced state of photosynthetic electron transport in illuminated thylakoid membranes and chloroplasts [33,34,35,36] and illuminated intact leaves of cucumber, Arabidopsis, and barley at lower temperature [37,38,39,40,41,42,43,44,45] and higher reduced state of P700 in angiosperms during rSP light illumination treatment [15,25,46,47,48], can cause PSI to be oxidatively damaged, depending on the presence of O_2_. The higher reduced state of Fe/S found in the present study reflected the reduction of phylloquinone, Fx, F_A_/F_B_, and Fd at the acceptor side of PSI. Reduced phylloquinone can donate electrons to O_2_ producing O_2_^−^ [5,12,13]. Fx, F_A_/F_B_, and Fd can reduce O_2_ to O_2_^−^ [5,11,12,13], which can oxidize the PSI.

However, regarding the kinetic interactions of these components with O_2_, the reduction of O_2_ to O_2_^−^ would not be easy because the lifetime of phylloquinone A_1_ is less than 20 ns, the lifetime of Fx is less than 50 ns, and the lifetime of F_A_/F_B_ is 500 ns to 100 ms [5], which is too short to react with O_2_. In fact, the reduction rate of O_2_ to O_2_^−^ in PSI, the Mehler reaction rate, ranged from 15 to 30 μmol O_2_^−^ mg Chl^−1^ h^−1^ [10,49]. Assuming that the ratio of P700 to Chl is 1:600 in thylakoid membranes, the half time of O_2_ reduction was estimated to be 150 to 300 ms [4], indicating that O_2_ would not have the opportunity to react with these electron carriers [4]. In fact, the O_2_ reduction rate in the Mehler reaction is negligible [50,51]. Ruuska et al. [50] showed no enhancement of the Mehler reaction, even in the reduced electron sinks of transgenic tobacco with reduced amounts of Rubisco.

Asada et al. reported the production of O_2_^−^ in the aprotic interior of thylakoid membranes [11]. In the experiments using thylakoid membranes, no electron acceptors for PSI were present, and the electron carriers A_1_, F_X,_ and F_A_/F_B_ were greatly reduced. However, if the lifetime of the reduced electron carriers A_1_, F_X,_ and F_A_/F_B_ were prolonged, they might react with O_2_. These hypotheses have been recently supported by the following facts: (1) phylloquinones in PSI particles isolated from *Chlamydomonas* could reduce O_2_ to O_2_^−^ [12,13], and (2) Fe/S clusters were the primary targets of PSI photoinhibition [33,34,35,36,52]. These conditions contributed to the accumulation of electrons at the acceptor side of PSI, which enhanced the interactions of these electron carriers with O_2_. Furthermore, no photosynthesis control functioned to oxidize the P700. Consequently, all these electron carriers were reduced.

In the present study, we demonstrated the reduction of Fe/S in the continuous illumination treatments of actinic light to *pgr5^hope1^* (Figure 4). The reduction state of Fe/S was greater than 40% (Figure 4). The higher reduction of Fe/S in *pgr5^hope1^* prolonged the lifetime of the reduced electron carriers (A_1_, F_X_, and F_A_/F_B_), which enhanced the reduction of O_2_ to O_2_^−^ within the PSI complex. Furthermore, compared to WT, *pgr5^hope1^* induces less luminal acidification of thylakoid membranes [53], thus prolonging the lifetime of the O_2_^−^ produced in the aprotic interior of thylakoid membranes. In contrast to aqueous conditions, protons are absent in the aprotic interior of thylakoid membranes [54,55]. Hence, O_2_^−^ cannot be protonated, its lifetime is enhanced, and O_2_^−^ is more likely to react with PSI proteins located within the thylakoid membranes. At the low pH of the luminal space of thylakoid membranes, O_2_^−^ can diffuse to the luminal face of the thylakoid membranes and easily react with the protons in the luminal space to dismutate to H_2_O_2_ and H_2_O, which prevents the interaction of O_2_^−^ with the electron carriers (A_1_, F_X,_ and F_A_/F_B_). If O_2_^−^ is produced by xanthine oxidase in the dark, PSI is oxidatively damaged [35]. The O_2_^−^ produced in the aqueous space easily accesses the PSI complex from the stromal side of the thylakoid membranes and can easily oxidize PSI [35]. That is, the acidification of the luminal space of thylakoid membranes contributes to both the oxidation of the electron carriers to suppress the production of O_2_^−^ and the rapid dismutation of O_2_^−^ produced in the PSI complex to H_2_O_2_ in the luminal space by supporting the diffusion direction of O_2_^−^ in the aprotic interior space of thylakoid membranes to the luminal space, which is reflected as the O_2_^−^ gradient from the production site to the luminal space. These observations were attributed to P700 oxidation. In addition, there is a positive relationship between P700 oxidation and the protection of PSI inactivation against the highly reduced state of Fe/S. These protection mechanisms are mainly driven by luminal acidification and photosynthetic control.

As described above, the rSP light illumination treatments decreased Fe/S in PSI (Figure 2, Figure 3 and Figure 5). The degradation of Fe/S clusters, including F_X_ and F_A_/F_B_ in the photo inactivated PSI has already been reported [36,39,52]. Furthermore, the degradation of PSI, PSI-A, and PSI-B reaction center proteins was observed under the photoinhibition of PSI [44,52], which was triggered by the O_2_^−^ produced in PSI. O_2_^−^ can inactivate the *Escherichia coli* enzymes dihydroxy-acid dehydratase, fumarase A, and fumarase B, as well as mammalian aconitase with rate constants ranging from 10^6^ to 10^7^ m^−1^ s^−1^ [56]. These enzymes have a 4Fe-4S cluster in their active sites. One of the irons in the reduced form of 4Fe-4S is attacked by O_2_^−^ with a negative free energy change (from −10 to −30 kcal/mol) and degraded to 3Fe-4S with the release of one Fe, which cannot catalyze the electron transfer reaction [56]. Simultaneously, H_2_O_2_ is formed by the attachment of O_2_^−^ to the 3Fe-4S cluster. Both Fe and H_2_O_2_ produced at the same site initiate the Fenton reaction to produce highly reactive ·OH, which might destroy the PSI-A/PSI-B polypeptide. In contrast, spinach dihydroxy-acid dehydratase containing a 2Fe-2S cluster in the active site showed resistance against O_2_^−^ attack [56]. That is, the degradation of Fe/S clusters by O_2_^−^ is specific to the 4Fe-4S clusters. In the present study, we observed a decrease in Fe/S amount in the leaves of *A. thaliana* WT, *pgr5^hope1^*, and *paa1*-*7*/*pox1* (Figure 2, Figure 3 and Figure 5). Fd contains a 2Fe-2S cluster in its catalytic center [57] and it could not be degraded by the photoinactivation treatment. If F_x_ and/or F_A_/F_B_ are degraded by O_2_^−^, Fd cannot accept electrons from the PSI to be reduced. The remaining Fd could not be measured in our assay system (see “Materials and Methods”). As a result, Fe/S decreased to approximately 30% after rSP illumination (Figure 2, Figure 3 and Figure 5).

**Figure 6:**
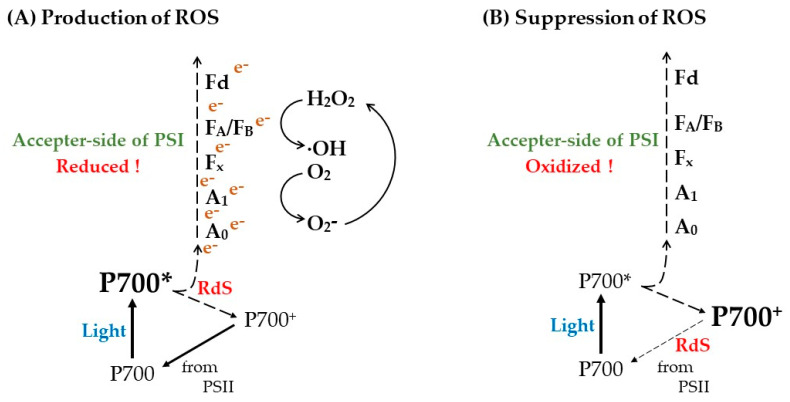
Production and suppression mechanism of ROS in PSI. PSI catalyzes the photosynthetic electron transport through a photo-oxidation reduction cycle in the reaction center of P700. P700 is photo-excited to P700* by the absorbed light energy and donates electrons to the primary electron acceptor chlorophyll *a* (A_0_). P700^+^ accepts electrons from PSII through plastoquinol, Cyt *b*6/*f*-complex, and plastocyanin (PC), regenerating P700. The electron in A_0_ flows to ferredoxin (Fd) through phylloquinone A_1_ and the iron-sulfur (4Fe-4S) clusters F_x_ and F_A_/F_B_. The accumulation of P700^+^ under the constant photon flux density is determined by the rate-determining step (RdS) of the production-consumption rate of P700^+^ in the photo-oxidation reduction cycle of P700 in PSI. At the RdS of P700*, oxidation P700^+^ does not accumulate; at the RdS of P700^+^ reduction, P700^+^ accumulates. (**A**) The RdS of P700* oxidation is caused by the accumulation of electrons at the acceptor-side of PSI, as observed with the reduction of Fe/S clusters (Fe/S^−^), including F_x_, F_A_/F_B_, and Fd, where the possibilities of the reduction of phylloquinone A_1_ also increase. These accumulated electrons would flow to O_2_ to produce superoxide radical (O_2_^−^), and O_2_^−^ would degrade F_X_ and F_A_/F_B_ with the release of Fe and hydrogen peroxide (H_2_O_2_). Both H_2_O_2_ and the reduced Fe further react to produce hydroxyl radical (OH) through Fenton reaction. This highly reactive oxygen species (ROS) would oxidatively degrade PSI irreversibly, leading to PSI inactivation. (**B**) The RdS of P700^+^ reduction is caused by the limitation of photosynthetic electron transport from plastoquinol to P700 through Cyt *b*6/*f*-complex and PC [46,48]. Even under low photosynthesis efficiency conditions (drought, high intensity light, low/high temperature, low CO_2_, etc.), P700 is oxidized to P700^+^. The RdS of P700^+^ reduction is induced by the acidification in the luminal side of thylakoid membranes, which suppress the oxidation activity of plastoquinol by the Cyt *b*6/*f*-complex, with the oxidation of the electron acceptors A_0_, A_1_, F_X_, F_A_/F_B_, and Fd, leading to the suppression of ROS production. This is the physiological function of P700 oxidation to suppress ROS production in PSI.

In the present study, we confirmed that the accumulation of electrons at the acceptor side of PSI, observed as the reduction of Fe/S, is the trigger of PSI inactivation. All oxygenic photosynthetic organisms oxidize P700 at low photosynthetic efficiency (e.g., under drought, fluctuating light, fluctuating stomata opening, high light, low temperature) [47,53,58,59,60,61,62,63,64,65,66]. Under these conditions, photosynthesis control driven by luminal acidification of thylakoid membranes downregulates the oxidation activity of plastoquinol in the Cyt *b*6/*f* complex [67]. Then, the rate-determining step (RdS) of the P700-photooxidation reduction cycle in PSI is shifted to the reduction reaction of oxidized P700 for the accumulation of P700^+^ [46,48]. Consequently, the reduction of Fe/S that leads to ROS production can be mitigated. This is the physiological function of P700 oxidation (Figure 6).

## Data Availability

Not applicable.
